# The Southern Illinois University Longitudinal Cognitive Aging Study: A Cohort Analysis

**DOI:** 10.1002/alz.087806

**Published:** 2025-01-09

**Authors:** Zachary Settelmyer, Mark S. Overby, Albert Botchway, Steven Scaife, Ronald F. Zec, Thomas A. Ala, Raj C. Shah, Eukesh Ranjit, Erin R. Hascup, Mehul A. Trivedi

**Affiliations:** ^1^ SIU School of Medicine, Springfield, IL USA; ^2^ Rush University Medical Center, Chicago, IL USA; ^3^ Southern Illinois University School of Medicine, Springfield, IL USA

## Abstract

**Background:**

The Southern Illinois University Longitudinal Cognitive Aging Study (SIU LCAS) is a community‐based, longitudinal cohort study initiated in 1984. LCAS was initially designed to improve the sensitivity of neuropsychological testing to diagnose mild cognitive impairment (MCI) and dementia in individuals residing in central and southern Illinois.

**Method:**

Participants are recruited from the community via newspaper advertisement, word‐of‐mouth, and community presentations. Individuals with a family history of Alzheimer’s dementia (AD) are strongly represented in this cohort. Participants must be cognitively normal at baseline and free from any neurological, uncontrolled medical, or psychiatric disease at their first visit. Recruitment is primarily focused on individuals over age 60. Participants complete a neuropsychological test battery assessing all major cognitive domains as well as questionnaires assessing personality, subjective cognitive ability, lifestyle factors, and detailed medical history.

**Result:**

The study has enrolled over 1,600 normal control participants since study inception. 150 original participants continue their participation. The average age at first visit is 66.5 (age range: 20‐95). The majority of participants are female (68.4%) and White/Not Hispanic (97.8%). Most participants (93%) have at least 12 years of education. 95% of the participants remained in the state of Illinois throughout their study participation. 29% of participants come from small or rural towns based on NCES locale boundaries data. 39% of participants reside in disadvantaged communities based on Area Deprivation Index scores of 8‐10. 55.4% of participants completed at least two study visits. 22% participants have completed >10 study visits. 63% of study participants are deceased. Only 26 of the deceased participants were enrolled in the brain donation program (17 with autopsy‐confirmed Alzheimer’s disease). 103 individuals met the diagnostic criteria for MCI or AD at baseline. 87 participants enrolled as controls declined to MCI or AD during their participation. 15 participants developed another neurological condition. An additional 86 participants who discontinued participation were found to have been diagnosed with MCI or dementia after discontinuation based on available medical records.

**Conclusion:**

Current directions involve the recruitment of participants from areas of central and southern Illinois who reside in some of the most disadvantages communities in the United States.

